# Supplementary material for: Diagnostic accuracy and limit of detection of ten malaria parasite lactate dehydrogenase-based rapid tests for Plasmodium knowlesi and P. falciparum
Source: Front Cell Infect Microbiol. 2022 Oct 17;12:1023219. doi: 10.3389/fcimb.2022.1023219 (PMC9618705; doi:10.3389/fcimb.2022.1023219)
Supplement: Supplementary file 1 [file Table_1.docx]

**Supplementary Table 1**. Comparison of sensitivity of RDT target antigens to detect *P. knowlesi* grouped by parasite count thresholds of **<**200 parasites/µL and ≥200 parasites/µL

| **RDT** | **Parasite count** | | | | | |
| --- | --- | --- | --- | --- | --- | --- |
|  | **<200 parasites/µL** | | | **≥200 parasites/µL** | | |
|  | **Pre-treatment**  **Sensitivity, n/N**  **% (95% CI)** | **Post-treatment**  **Sensitivity, n/N**  **% (95% CI)** | **p-value** | **Pre-treatment**  **Sensitivity, n/N**  **% (95% CI)** | **Post-treatment**  **Sensitivity, n/N**  **% (95% CI)** | **p-value** |
| **Pan-pLDH antigen** | | | | | | |
| First Response® | 3/7  **42.9**  (9.9 – 81.6) | 10/20  **50.0**  (27.2 – 72.8) | 0.74 | 44/47  **93.6**  (82.5 – 98.7) | 28/29  **96.6**  (82.2 – 99.9) | 0.58 |
| CareStart™ PAN | 3/7  **42.9**  (9.9 – 81.6) | 10/20  **50.0**  (27.2 – 72.8) | 0.74 | 44/47  **93.6**  (82.5 – 98.7) | 29/29  **100**  (88.1 – 100) | 0.17 |
| Standard Q | 7/17  **41.2**  (18.4 – 67.1) | 7/20  **35.0**  (15.4 – 59.2) | 0.70 | 66/69  **95.7**  (87.8 – 99.1) | 35/37  **94.6**  (81.8 – 99.3) | 0.81 |
| careStart^TM^ combo | 2/6  **33.3**  (4.33 – 77.7) | 7/20  **35.0**  (15.4 – 59.2) | 0.94 | 42/47  **89.4**  (76.9 – 96.5) | 27/29  **93.1**  (77.2 – 99.2) | 0.58 |
| Parascreen | 1/6  **16.7**  (0.42 – 64.1) | 2/20  **10.0**  (1.23 – 31.7) | 0.65 | 39/47  **83.0**  (69.2 – 92.4) | 25/29  **86.2**  (68.3 – 96.1) | 0.71 |
| CareUS™ | 1/16  **6.25**  (0.16 – 30.2) | 2/19  **10.5**  (1.30 – 33.1) | 0.65 | 51/64  **79.7**  (67.8 – 88.7) | 25/34  **73.5**  (55.6 – 87.1) | 0.49 |
| SD Bioline | 0/16  **0**  (0 – 0) | 1/21  **4.76**  (0.12 – 23.8) | 0.38 | 44/71  **62.0**  (49.7 – 73.2) | 16/37  **43.2**  (27.1 – 60.5) | 0.06 |
| ***Pv*-pLDH antigen** | | | | | | |
| Biocredit™ | 14/19  **73.7**  (48.8 – 90.9) | 15/17  **88.2**  (63.6 – 98.5) | 0.27 | 67/69  **97.1**  (89.9 – 99.6) | 30/30  **100.0**  (88.4 – 100) | 0.35 |
| FalciVax | 9/18  **50.0**  (26.0 – 74.0) | 12/21  **57.1**  (34.0 – 78.2) | 0.66 | 69/72  **95.8**  (88.3 – 99.1) | 26/27  **96.3**  (81.0 – 99.9) | 0.92 |

**Supplementary Table 2.** Spearman’s correlation test between proportion of early trophozoite (ring) stages to RDT test positivity.

| RDT | Observations | Spearman’s rho | p-value |
| --- | --- | --- | --- |
| Pan-pLDH antigen | | | |
| First Response® | 54 | -0.194 | 0.160 |
| careStart™ PAN | 54 | -0.569 | <0.001 |
| Standard Q | 86 | -0.206 | 0.057 |
| careStart™ Combo | 53 | -0.477 | 0.0003 |
| Parascreen | 53 | -0.123 | 0.382 |
| CareUS™ | 80 | -0.144 | 0.204 |
| SD Bioline | 87 | -0.176 | 0.103 |
| *Pv*-pLDH antigen | | | |
| Biocredit™ | 88 | -0.322 | 0.002 |
| FalciVax | 90 | -0.129 | 0.227 |

**Supplementary Table 3.** Comparative sensitivity (pre vs post antimalarial treatment) of RDT main target antigens in detecting *P. knowlesi* from clinical samples

| RDT | Median time post treatment in hours (IQR)^b^ | Pre-treatment Sensitivity, n/N  % (95% CI) | Post-treatment Sensitivity , n/N  % (95% CI) | Relative difference % (p-value) | Specificity ^a^  n/N  % (95% CI) | |
| --- | --- | --- | --- | --- | --- | --- |
| Pan-pLDH antigen | | | | | |  |
| First Response® | 2.84  (1.73 – 7.09) | 47/54  **87.0**  (75.1 – 94.6) | 38/49  **77.6**  (63.4 – 88.2) | 9.4  (0.21) | 19/19  **100**  (82.4 – 100) | |
| careStart™ PAN | 2.84  (1.73 – 7.09) | 47/54  **87.0**  (75.1 – 94.6) | 39/49  **79.6**  (65.7 – 89.8) | 7.4  (0.31) | 19/19  **100**  (82.4 – 100) | |
| Standard Q | 3.00  (1.52 – 4.36) | 73/86  **84.9**  (75.5 – 91.7) | 42/57  **73.7**  (60.3 – 84.5) | 11.2  (0.10) | 21/21  **100**  (83.9 – 100) | |
| careStart™ Combo | 2.84  (1.73 – 7.09) | 44/53  **83.0**  (70.2 – 91.9) | 34/49  69.4  (54.6 – 81.7) | 13.6  (0.10) | 19/19  **100**  (82.4 – 100) | |
| Parascreen | 2.84  (1.73 – 7.09) | 40/53  **75.5**  (61.7 – 86.2) | 27/49  **55.1**  (40.2 – 69.3) | **20.4**  **(0.03)** | 19/19  **100**  (82.4 – 100) | |
| CareUS™ | 3.33  (1.73 – 5.47) | 52/80  **65.0**  (53.5 – 75.3) | 27/53  **50.9**  (36.8 – 64.9) | 14.1  (0.11) | 21/21  **100**  (83.9 – 100) | |
| SD Bioline | 2.92  (1.73 – 4.36) | 44/87  **50.6**  (39.6 – 61.5) | 17/58  **29.3**  (18.1 – 42.7) | **21.3**  **(0.01)** | 22/22  **100**  (84.6 – 100) | |
| *Pv*-pLDH | | | | | |  |
| Biocredit™ | 3.58  (1.96 – 7.54) | 81/88  **92.0**  (84.3 – 96.7) | 45/47  **95.7**  (85.5 – 99.5) | 3.7  (0.41) | 20/20  **100**  (83.2 – 100) | |
| FalciVax | 3.51  (1.49 – 6.30) | 78/90  **86.7**  (77.9 – 92.9) | 38/48  **79.2**  (65.0 – 89.5) | 7.5  (0.25) | 18/18  **100**  (81.5 – 100) | |

^a^No difference in specificity for pre- and post- antimalarial group testing.

^b^Median time post-treatment between the RDTs conducted were not significant (*X*^2^ (8, N = 9) = 2.58, p = .96).
